# Association of *ATP2B1* and *STK39* gene variants with blood pressure levels in patients with essential hypertension

**DOI:** 10.1093/labmed/lmag025

**Published:** 2026-05-20

**Authors:** Egemen Akgün, Ahmet Karagöz, Fadime Mutlu İçduygu, Ebru Alp

**Affiliations:** Department of Medical Biology, Faculty of Medicine, Giresun University, Giresun, Turkey; Department of Cardiology, Faculty of Medicine, Samsun University, Samsun, Turkey; Department of Medical Genetics, Faculty of Medicine, Giresun University, Giresun, Turkey; Department of Medical Biology, Faculty of Medicine, Giresun University, Giresun, Turkey

**Keywords:** *ATP2B1*, *STK39*, gene variant, essential hypertension, blood pressure, sex-specific effect

## Abstract

**Introduction:**

*ATP2B1* and *STK39* loci influence blood pressure in genome-wide association studies. We tested whether *ATP2B1* rs2681472 and *STK39* rs35929607 are associated with essential hypertension and blood pressure.

**Methods:**

We studied 194 untreated hypertensive adults and 191 normotensive control individuals. Genotypes were determined by polymerase chain reaction–restriction fragment length polymorphism. Logistic regression evaluated hypertension risk, and linear regression assessed systolic and diastolic blood pressure (BP) under genotypic, dominant, and additive models, with adjustment for clinical covariates; the false discovery rate was controlled.

**Results:**

The *STK39* rs35929607 G allele was associated with hypertension (adjusted odds ratio [OR], 1.97 [95% CI, 1.21-3.21]; *P* = .007) and higher systolic BP (+8.02 mm Hg per G allele, 95% CI, 2.18-13.87; *P* = .007). Effects on diastolic BP were weaker (additive *P* = .10; dominant *P* = .06). *ATP2B1* rs2681472 showed no statistically significant associations (hypertension per T allele OR, 1.18 [95% CI, 0.74-1.87]; *P* = .49). In sex-stratified analyses, *STK39* associations were evident in men (hypertension OR, 3.22 [95% CI, 1.42-7.31]; *P* = .005; systolic BP, +15.88 mm Hg [95% CI, 5.95-25.80]; *P* = .002) but not in women.

**Discussion:**

In our study cohort, *STK39* rs35929607 is associated with essential hypertension and higher systolic BP, with possible sex-specific effects. *ATP2B1* rs2681472 is not associated with hypertension or BP traits.

## Introduction

Hypertension is a sustained elevation of systemic arterial blood pressure (BP) and a leading modifiable cause of stroke, myocardial infarction, heart failure, and chronic kidney disease. Clinically, hypertension is classified as primary (essential) when no secondary cause is identified and secondary when a specific etiology is present, which should be considered particularly in younger patients.^1^ Although estimates vary by region, hypertension affects a substantial share of adults worldwide and remains a leading public health problem.^2^

Interindividual variation in BP has a substantial heritable component, and genome-wide association studies (GWASs) have transformed our understanding of its polygenic architecture.^3^^,^^4^ Early GWASs from the Cohorts for Heart and Aging Research in Genomic Epidemiology (CHARGE) consortium and others identified multiple common loci that influence systolic and diastolic BPs and hypertension risk, establishing a framework that has since expanded dramatically with large meta-analyses.^5^^,^^6^ Most recently, a more than 1 million–participant study reported hundreds of loci associated with BP traits, highlighting pathways in vascular tone, renal salt handling, and endocrine regulation.^3^ Within this body of work, signals at or near *ATP2B1* and *STK39* have been among the most consistently observed.^3^^,^^5–7^

*ATP2B1* encodes plasma membrane calcium ATPase 1 (PMCA1), a ubiquitous calcium ion (Ca^2+^) efflux pump that helps maintain low cytosolic calcium in many tissues, including vascular smooth muscle.^8^ Reduced PMCA1 activity is expected to raise intracellular Ca^2+^, increase small-artery constrictor tone, and elevate BP.^9^^,^^10^ Human genetic associations at *ATP2B1* are consistent with this mechanism, and earlier molecular studies of PMCA1 support a causal link between impaired calcium extrusion and hypertension.^3^^,^^5^^,^^11^

*STK39* encodes Ste20-related proline-alanine–rich kinase (SPAK), a serine-threonine kinase that transduces WNK-dependent signals to transporters governing renal sodium reabsorption.^12^^,^^13^ When activated downstream of WNK1 and WNK4, SPAK phosphorylates the loop diuretic–sensitive Na^+^-K^+^-2Cl^-^ co-transporter, abbreviated NKCC2, and the thiazide-sensitive Na^+^-Cl^-^ co-transporter, abbreviated NCC.^13^^,^^14^ Modulation of these transporters shifts tubular sodium handling and extracellular volume, which in turn affects blood pressure.^12^^,^^15^ Discovery and follow-up studies have linked common variation at *STK39* to hypertension susceptibility in several populations, consistent with its central role in distal nephron physiology.^7^^,^^11^

At the variant level, *ATP2B1* rs2681472 (C>T) emerged as an index signal at the *ATP2B1* locus in large GWASs/meta-analyses of BP, including the CHARGE/Global BPgen framework, and has been associated with systolic BP and diastolic BP across multiple cohorts.^5^^,^^6^ Subsequent meta-analyses further supported the association of rs2681472 with hypertension susceptibility and BP levels, albeit with modest effect sizes.^11^ Biological plausibility is strengthened by experimental evidence showing that vascular smooth muscle cell–specific loss of *ATP2B1* increases BP and alters intracellular Ca^2+^ handling and vasoconstrictive responses.^9^ Similarly, *STK39* was identified as a hypertension susceptibility gene in a GWAS, and multiple *STK39* variants have demonstrated association signals with BP in both the discovery and replication settings.^7^ Subsequent systematic reviews and meta-analyses across diverse populations have evaluated several common intronic *STK39* polymorphisms—most consistently rs3754777, rs35929607, and rs6749447—supporting a modest and context-dependent contribution of this locus to essential hypertension susceptibility and underscoring between-study heterogeneity; moreover, rs3754777 has been reported to be in strong linkage disequilibrium with rs35929607, which strengthens the rationale for focusing on rs35929607 in our cohort.^16^^,^^17^ In middle-aged Swedish people from the Malmö cohorts (Sweden), rs35929607 was evaluated in relation to BP and hypertension, and after stratification by sex the association remained statistically significant in women but not in men, suggesting potential sex-dependent effects.^18^

Given these prior findings and reported population-related and sex-related heterogeneity, we investigated whether *ATP2B1* rs2681472 and *STK39* rs35929607 are associated with essential hypertension susceptibility and clinic systolic BP/diastolic BP levels in our clinically characterized adult cohort; we additionally explored sex-stratified effects and secondary cardiovascular phenotypes to enhance clinical interpretability. To the best of our knowledge, this is the first study to evaluate these 2 variants in a Turkish essential hypertension case-control cohort.

## Methods

### Study population

This case-control study was conducted at the Cardiology Clinic of Giresun University Faculty of Medicine. The final sample comprised 194 patients with essential hypertension and 191 healthy control individuals without a history of hypertension. To minimize population stratification, case and control individuals were recruited from the same geographic catchment area at a single center, and related individuals were excluded. All participants were self-reported ethnic Turkish. Eligibility for the case group required newly diagnosed essential hypertension, age 18 years or older, absence of secondary hypertension, and no prior coronary heart disease, stroke, kidney failure, or diabetes. At the time of enrollment, no hypertensive patients had received any treatment for hypertension; the use of antihypertensive medication, either currently or previously, was an exclusion criterion. Control individuals had no history of hypertension, diabetes, ischemic heart disease, chronic kidney disease, or stroke. The study was approved by the Clinical Research Ethics Committee of Giresun University with decision No. 05.12.2019/24. The study complied with the Declaration of Helsinki, and written informed consent was obtained from all participants.

### Genotyping of rs2681472 and rs35929607

Genomic DNA was isolated from peripheral blood using a commercial kit from Roche Diagnostics. Genotyping was performed by polymerase chain reaction–restriction fragment length polymorphism. For rs2681472 in *ATP2B1*, the forward primer was 5′-TCTGAGGATGTGGCATTTGA-3′ and the reverse primer was 5′-CCCAGTAAGGCCAGAGATCA-3′. For rs35929607 in *STK39*, the forward primer was 5′-AGGGAGAAACATGTCCCAGTG-3′ and the reverse primer was 5′-GCCTGATTTGCCAGGCTTCT-3′. The polymerase chain reaction comprised 35 cycles with denaturation at 94 °C for 30 seconds, annealing at 56 °C for 45 seconds, and extension at 72 °C for 45 seconds. The *ATP2B1* amplicon was 203 base pairs (bp) and was digested with PvuII; the C allele created a restriction site yielding 141-bp and 62-bp fragments, while the T allele abolished the site and remained as an undigested 203-bp fragment, so CC yielded 141-bp and 62-bp fragments; CT yielded 203-bp, 141-bp, and 62-bp fragments; and TT yielded 203-bp fragments. The *STK39* amplicon was 356 bp and was digested with MspI; the G allele created a restriction site yielding 206-bp and 150-bp fragments, while the A allele abolished the site and remained as an undigested 356-bp fragment, so GG yielded 206-bp and 150-bp fragments; AG yielded 356-bp, 206-bp, and 150-bp fragments; and AA yielded 356-bp fragments.

### Statistical analysis

Continuous variables were summarized as mean (SD) and categorical variables as counts (percentages). Between-group comparisons (patients with essential hypertension vs control individuals) used the independent-samples *t* test (or the Mann-Whitney *U* test for non-normally distributed variables) for continuous variables and the χ^2^ test or Fisher exact test for categorical variables. Hardy-Weinberg equilibrium (HWE) in the control group was assessed for each single-nucleotide variation (formerly single-nucleotide polymorphism) using the Pearson χ^2^ test and the exact mid-*P* test. The primary outcome was hypertension status. For each single-nucleotide variation, odds ratios (ORs) and 95% CIs were estimated by logistic regression under genotypic, dominant, and additive (per-allele) genetic models. Multivariable models were adjusted for age (continuous), sex, body mass index, smoking status, fasting plasma glucose, creatinine, low-density lipoprotein cholesterol, high-density lipoprotein cholesterol, triglycerides, and thyrotropin (formerly thyroid-stimulating hormone). Systolic and diastolic BP were analyzed in the combined sample using multivariable linear regression with additive and dominant genotype terms and the same set of covariates. The predefined secondary traits (pulse wave velocity, augmentation index, and echocardiographic parameters) were analyzed using the same multivariable linear regression framework. To account for multiple testing in the primary case-control genetic comparisons and the analyses of secondary traits, we controlled the false discovery rate using the Benjamini-Hochberg procedure. All tests were 2 sided, with *P* < .05 (and, where applicable, *q* < 0.05) considered statistically significant. Exact and mid-*P* HWE *P* values were computed using the HardyWeinberg package in R (R Foundation for Statistical Computing), whereas all other analyses were performed in IBM SPSS Statistics, version 25, software. This case-control study is reported in accordance with the Strengthening the Reporting of Observational Studies in Epidemiology guidelines for observational studies.

## Results

### Study population

We enrolled 385 participants (194 essential hypertension cases; 191 control individuals). Compared with control individuals, case individuals had higher body mass index (mean [SD], 30.17 [4.82] vs 27.97 [4.68]; *P* < .001) and markedly elevated office systolic BP and diastolic BP (mean [SD] systolic BP, 171.60 [18.36] vs 122.24 [10.40] mm Hg; diastolic BP, 99.75 [12.43] vs 73.82 [8.96] mm Hg; both *P* < .001). Arterial stiffness (pulse wave velocity) and several echocardiographic structural indices were higher in cases (eg, mean [SD] pulse wave velocity, 8.03 [1.88] vs 7.27 [1.09] m/s, *P* < .001; left ventricular mass index, 92.56 [28.30] vs 69.00 [17.29] g/m^2^, *P* < .001; interventricular septal thickness, 1.04 [0.20] vs 0.86 [0.15] cm, *P* < .001), while left ventricular ejection fraction did not differ (mean [SD] left ventricular ejection fraction, 64.85% [2.71%] vs 65.00% [2.86%], *P* = .57). Routine laboratory markers were broadly similar, with modest case-control differences for fasting plasma glucose (mean [SD], 104.75 [22.58] vs 100.49 [15.51] mg/dL, *P* = .002), urea (31.80 [12.08] vs 26.70 [7.20] mg/dL, *P* < .001), serum creatinine (0.81 [0.18] vs 0.76 [0.16] mg/dL, *P* = .007), total cholesterol (208.65 [40.86] vs 200.76 [36.63] mg/dL, *P* = .02), triglycerides (168.69 [102.11] vs 143.25 [73.02] mg/dL, *P* = .02), and low-density lipoprotein cholesterol (129.60 [35.63] vs 121.84 [32.50] mg/dL, *P* = .007), whereas high-density lipoprotein cholesterol (*P* = .07) and thyrotropin (*P* = .91) did not differ significantly. The baseline demographic, clinical, echocardiographic, and laboratory characteristics of the study population are summarized in Table 1.

**Table 1:** Demographic, clinical, echocardiographic, and laboratory characteristics of patients with essential hypertension and control individuals.

Parameter	**Case individuals *n*** = **194**	**Control individuals *n*** = **191**	*P* value^a^
Age, mean (SD), y	52.99 (9.36)	51.08 (8.83)	.04
Age range, y	28-73	31-78	
Age group, No. (%)			.68
<40 y	15 (7.7)	17 (8.9)	
≥40 y	179 (92.3)	174 (91.1)	
Sex, No. (%)			
Female	105 (54.1)	114 (59.7)	.27
Male	89 (45.9)	77 (40.3)	
Body mass index, mean (SD)	30.17 (4.82)	27.97 (4.68)	<.001
Clinic blood pressure, mean (SD), mm Hg			
Systolic	171.60 (18.36)	122.24 (10.40)	<.001
Diastolic	99.75 (12.43)	73.82 (8.96)	<.001
Arterial stiffness, mean (SD)			
Pulse wave velocity, m/s	8.03 (1.88)	7.27 (1.09)	<.001
Augmentation index, %	23.33 (28.16)	23.89 (12.82)	.02
Echocardiography—structure, mean (SD)			
Left ventricular mass index, g/m²	92.56 (28.30)	69.00 (17.29)	<.001
Interventricular septal thickness, cm	1.04 (0.20)	0.86 (0.15)	<.001
Left ventricular posterior wall thickness, cm	1.04 (0.22)	0.87 (0.13)	<.001
Left ventricular end-diastolic diameter, cm	4.70 (0.44)	4.55 (0.48)	<.001
Left ventricular end-systolic diameter, cm	2.66 (0.40)	2.63 (0.38)	.21
Aortic root diameter, cm	3.10 (0.39)	2.98 (0.33)	.005
Ascending aorta diameter, cm	3.16 (0.45)	2.92 (0.41)	<.001
Left atrial diameter, cm	3.17 (0.42)	3.00 (0.42)	<.001
Right ventricular diameter, cm	2.42 (0.29)	2.38 (0.30)	.01
Echocardiography—function, mean (SD)			
Left ventricular ejection fraction, %	64.85 (2.71)	65.00 (2.86)	.57
E/Ea	7.52 (4.91)	10.01 (5.83)	<.001
Laboratory—core cardiometabolic, mean (SD)			
Fasting plasma glucose, mg/dL	104.75 (22.58)	100.49 (15.51)	.002
Urea, mg/dL	31.80 (12.08)	26.70 (7.20)	<.001
Serum creatinine, mg/dL	0.81 (0.18)	0.76 (0.16)	.007
Total cholesterol, mg/dL	208.65 (40.86)	200.76 (36.63)	.02
Triglycerides, mg/dL	168.69 (102.11)	143.25 (73.02)	.02
High-density lipoprotein cholesterol, mg/dL	48.57 (12.89)	50.52 (13.99)	.07
Low-density lipoprotein cholesterol, mg/dL	129.60 (35.63)	121.84 (32.50)	.007
Thyrotropin, mIU/L	2.12 (1.94)	2.42 (5.65)	.91

^a^ *P* < .05 was considered statistically significant.

### Genotyping quality and allele frequencies

Control groups were in HWE for both loci (*STK39* rs35929607: χ^2^  *P* = .94, exact mid-*P P* = .91; *ATP2B1* rs2681472: χ^2^  *P* = .55, exact mid-*P P* = .64), as shown in Supplementary Table 1. Minor-allele frequencies were 0.19 in control individuals and 0.28 in case individuals for *STK39* and 0.26 in control individuals and 0.28 in case individuals for *ATP2B1*. Detailed genotype and allele distributions in case and control individuals are shown in Table 2.

**Table 2:** Genotype and allele distributions of *STK39* rs35929607 (A>G) and *ATP2B1* rs2681472 (C>T) in patients with essential hypertension and control individuals.

Genotype	**Case, No. (%) *n*** = **194**	**Control, No. (%) *n*** = **191**	*P* value^a^	OR (95% CI)^b^	*P* value^a^	AOR (95% CI)^b^^,^^c^	Adjusted *P* value^c^	Benjamini-Hochberg false discovery rate, *q*
*STK39*			.02					
AA	100 (51.5)	124 (64.9)		1				
AG	81 (41.8)	60 (31.4)		1.64 (1.09-2.56)	.02	1.90 (1.08-3.35)	.03	0.04
GG	13 (6.7)	7 (3.7)		2.30 (0.89-5.99)	.08	5.16 (1.01-26.32)	.049	0.049
AG + GG	94 (48.5)	67 (35.1)		1.74 (1.16-2.62)	.008	2.06 (1.19-3.58)	.01	0.02
Alleles			.007					
A	281 (72.4)	308 (80.6)		1				
G	107 (27.6)	74 (19.4)		1.59 (1.13-2.22)	.007	1.97 (1.21-3.21)	.007	0.02
*ATP2B1*			.66					
CC	97 (50.0)	104 (54.5)		1				
CT	86 (44.3)	76 (39.8)		1.21 (0.80-1.84)	.36	1.19 (0.68-2.09)	.54	0.65
TT	11 (5.7)	11 (5.8)		1.07 (0.45-2.59)	.88	1.36 (0.36-5.06)	.65	0.65
CT + TT	97 (50.0)	87 (45.5)		1.20 (0.80-1.78)	.38	1.21 (0.70-2.07)	.50	0.65
Alleles			.49					
C	280 (74.3)	284 (72.2)		1				
T	108 (25.7)	98 (27.8)		1.12 (0.81-1.54)	.49	1.18 (0.74-1.87)	.49	0.65

Abbreviations: AOR, adjusted odds ratio; OR, odds ratio.

^a^ *P* values refer to overall genotype or allele distribution tests (Pearson χ^2^).

^b^ Odds ratios and 95% CIs were estimated using logistic regression.

^c^ Adjusted models included age, sex, body mass index, smoking status, fasting plasma glucose, low-density lipoprotein cholesterol, high-density lipoprotein cholesterol, triglycerides, serum creatinine, and thyrotropin.

### Case-control genetic association (primary case-control analysis)

For *STK39* rs35929607 (A>G), the genotypic test was statistically significant (*P* = .02). In covariate-adjusted models, AG vs AA yielded an OR of 1.90 (95% CI, 1.08-3.35; *P* = .03; Benjamini-Hochberg false discovery rate *q* = 0.04), and the rare GG genotype also showed higher odds of hypertension compared with AA, although with a very imprecise estimate (OR, 5.16 [95% CI, 1.01-26.32]; *P* = .049; *q* = 0.049). A dominant comparison of carriers (AG + GG) vs noncarriers (AA) was likewise statistically significant (adjusted OR [AOR], 2.06 [95% CI, 1.19-3.58]; *P* = .01; *q* = 0.02), and the allelic (per G allele) model yielded an OR of 1.97 (95% CI, 1.21-3.21; *P* = .007; *q* = 0.02) (Table 2). In contrast, *ATP2B1* rs2681472 (C>T) showed no association with hypertension: CT vs CC OR, 1.19 (95% CI, 0.68-2.09; *P* = .54; *q* = 0.65), TT vs CC OR, 1.36 (95% CI, 0.36-5.06; *P* = .65; *q* = 0.65), dominant OR, 1.23 (95% CI, 0.70-2.07; *P* = .50; *q* = 0.65), and allelic OR, 1.18 (95% CI, 0.74-1.87; *P* = .49; *q* = 0.65).

In sex-stratified analyses, the association between rs35929607 and hypertension appeared to be restricted to men. Among men, the additive model (per G allele) remained independently associated with hypertension after multivariable adjustment (AOR, 3.22 [95% CI, 1.42-7.31]; *P* = .005), and the dominant comparison (AG + GG vs AA) showed a similar increase in risk (OR, 3.23 [95% CI, 1.39-7.53]; *P* = .007). In women, neither the additive nor the dominant model was statistically significant in the adjusted logistic regression (additive *P* = .25; dominant *P* = .37), and in the adjusted logistic model, the *STK39* additive term was null (Table 3). For *ATP2B1* rs2681472, no evidence of association with hypertension was observed in either sex.

**Table 3:** Sex-stratified association between *STK39* rs35929607 and hypertension.

Sex	Genetic model	Case/control,^a^ No.	AOR (95% CI)^b^	Adjusted *P* value^c^
Male	Additive	89/77	3.22 (1.42-7.31)	.005
	Dominant	89/77	3.23 (1.39-7.53)	.007
Female	Additive	105/114	1.47 (0.77-2.80)	.25
	Dominant	105/114	1.43 (0.66-3.10)	.37

Abbreviation: AOR, adjusted odds ratio.

^a^ Indicates the number of hypertensive case individuals and normotensive control individuals included in each sex-specific model.

^b^ Adjusted odds ratios with 95% CIs and *P* values are derived from multivariable logistic regression under additive and dominant genetic models. In the additive model, the AOR represents the change in hypertension odds per minor (G) allele of *STK39* rs35929607; in the dominant model, the AOR compares carriers (AG + GG) with noncarriers (AA). Models were adjusted for age, body mass index, smoking status, fasting plasma glucose, creatinine, low-density lipoprotein cholesterol, high density lipoprotein cholesterol, triglycerides, and thyrotropin.

^c^ Two-sided *P* < .05 was considered statistically significant.

### Quantitative BP (primary quantitative analysis)

In multivariable linear regression across the full cohort, *STK39* rs35929607 was positively associated with systolic BP under both genetic codings. In the additive model, each additional G allele was associated with an increase of 8.02 mm Hg in systolic BP (95% CI, 2.18-13.87; *P* = .007), and in the dominant model (AG + GG vs AA), systolic BP was higher by 9.54 mm Hg (95% CI, 2.65-16.44; *P* = .007). For diastolic BP, the corresponding per-allele effect was β = +2.88 mm Hg (95% CI, −0.56 to 6.32; *P* = .10) and β = +3.94 mm Hg in the dominant model (95% CI, −0.09 to 8.00; *P* = .06). For *ATP2B1* rs2681472, additive per-allele effects on systolic and diastolic BP were small and nonsignificant (systolic BP β = +2.91 mm Hg [95% CI, −3.01 to 8.82]; *P* = .34; diastolic BP β = +1.60 mm Hg [95% CI, −1.85 to 5.06]; *P* = .36), and dominant-model estimates were likewise null (systolic BP β = +3.63 mm Hg [95% CI, −3.33 to 10.58]; *P* = .30; diastolic BP β = +1.23 mm Hg [95% CI, −2.84 to 5.30]; *P* = .55). Genotype-stratified mean systolic and diastolic BP values and the corresponding multivariable regression coefficients for *STK39* and *ATP2B1* are summarized in Table 4.

**Table 4:** Office BP by *STK39* rs35929607 and *ATP2B1* rs2681472 genotypes, with multivariable association estimates in the total study population.

rs35929607^a^	AA, mean (SD) *n* = 224	AG, mean (SD) *n* = 141	GG, mean (SD) *n* = 20	AG + GG, mean (SD) *n* = 161	Additive β (95% CI)^b^	Adjusted *P* value (additive)^b^	Dominant β (95% CI)^b^	Adjusted *P* value (dominant)^b^
Systolic BP, mm Hg	143.10 (28.02)	150.04 (30.92)	154.80 (29.30)	150.65 (30.67)	8.02 (2.18-13.87)	.007	9.54 (2.65-16.44)	.007
Diastolic BP, mm Hg	84.48 (15.98)	88.43 (16.69)	85.90 (19.32)	88.10 (16.98)	2.88 (‒0.56 to 6.32)	.10	3.94 (‒0.09 to 8.00)	.06

rs2681472^a^	CC, mean (SD) *n* = 201	CT, mean (SD) *n* = 162	TT, mean (SD) *n* = 22	CT + TT, mean (SD) *n* = 184	Additive β (95% CI)	Adjusted *P* value (additive)	Dominant β (95% CI)	Adjusted *P* value (dominant)
Systolic BP, mm Hg	145.01 (29.87)	148.55 (28.59)	140.10 (30.03)	147.57 (28.80)	2.91 (‒3.01 to 8.82)	.34	3.63 (‒3.33 to 10.58)	.30
Diastolic BP, mm Hg	85.52 (16.54)	86.62 (16.31)	85.57 (17.75)	86.49 (16.44)	1.60 (‒1.85 to 5.06)	.36	1.23 (‒2.84 to 5.30)	.55

Abbreviation: BP, blood pressure.

^a^ Values are reported by genotype (*STK39* rs35929607 A>G: AA/AG/GG; *ATP2B1* rs2681472 C>T: CC/CT/TT) and for the dominant grouping (*STK39:* AG + GG vs AA; *ATP2B1:* CT + TT vs CC).

^b^ Coefficients (β, mm Hg) with 95% CI and *P* values are from multivariable linear regression. The additive model reports the effect per minor allele (*STK39:* per G; *ATP2B1:* per T); the dominant model compares carriers with noncarriers. Models were adjusted for age, sex, body mass index, smoking status, fasting plasma glucose, creatinine, low-density lipoprotein cholesterol, high-density lipoprotein cholesterol, triglycerides, and thyrotropin; case status was not included as a covariate. Two-sided *P* < .05 was considered statistically significant.

When these models were stratified by sex, the *STK39*-BP relationship was again restricted to men. In men, each additional G allele of rs35929607 was associated with markedly higher BP (systolic BP β = +15.88 mm Hg [95% CI, 5.95-25.80]; *P* = .002; diastolic BP β = +9.04 mm Hg [95% CI, 2.92-15.17]; *P* = .004), whereas in women the per-allele coefficients were small and nonsignificant (systolic BP β = +3.22 mm Hg, *P* = .40; diastolic BP β = −1.09 mm Hg, *P* = .61). For *ATP2B1* rs2681472, sex-stratified models also showed no clear association with BP. In men, the dominant *ATP2B1* term (CT + TT vs CC) yielded β = +0.18 mm Hg for systolic BP (95% CI, −11.51 to 11.88; *P* = .98) and β = +1.95 mm Hg for diastolic BP (95% CI, −5.20 to 9.11; *P* = .59). In women, the additive *ATP2B1* term showed β = +3.82 mm Hg per T allele for systolic BP (95% CI, −3.57 to 11.20; *P* = .31) and β = +0.67 mm Hg for diastolic BP (95% CI, −3.41 to 4.75; *P* = .75) (Table 5).

**Table 5:** Sex-stratified associations between systolic/diastolic BP and *STK39* rs35929607.

Male	Case/control,^a^ No.	Additive β (95% CI)^b^	Adjusted *P* value (additive)^b^	Dominant β (95% CI)^b^	Adjusted *P* (dominant)^b^
Systolic BP, mm Hg	89/77	15.88 (5.95-25.80)	.002	17.66 (6.70-28.62)	.002
Diastolic BP, mm Hg	89/77	9.04 (2.92-15.17)	.004	9.88 (3.11-16.66)	.005

Female	Case/control,^a^ No.	Additive β (95% CI)^b^	Adjusted *P* value (additive)^b^	Dominant β (95% CI)^b^	Adjusted *P* value (dominant)^b^
Systolic BP, mm Hg	105/114	3.22 (‒4.37 to 10.80)	.40	3.10 (‒6.24 to 12.44)	.51
Diastolic BP, mm Hg	105/114	‒1.09 (−5.28 to 3.09)	.61	‒1.08 (‒6.23 to 4.08)	.68

Abbreviation: BP, blood pressure.

^a^ Indicates the number of hypertensive case individuals and normotensive control individuals contributing to each sex-specific model.

^b^ Regression coefficients (β, mm Hg) with 95% CIs and *P* values are from multivariable linear regression under additive and dominant genetic models. In the additive model, β represents the change in BP per minor (G) allele; in the dominant model, β compares carriers (AG + GG) with noncarriers (AA). Models were adjusted for age, body mass index, smoking status, fasting plasma glucose, creatinine, low-density lipoprotein cholesterol, high-density lipoprotein cholesterol, triglycerides, and thyrotropin. Two-sided *P* < .05 was considered statistically significant.

### Secondary cardiovascular traits

For *STK39*, none of the arterial stiffness or echocardiographic traits (pulse wave velocity, augmentation index, left ventricular structure, and function indices) showed evidence of association after covariate adjustment; when the secondary outcome set was false discovery rate controlled, no *q* < 0.05 were observed, as summarized in Table 6.

**Table 6:** Association between *STK39* rs35929607 genotypes and clinical characteristics in the total study population.

Characteristic	**AA *n*** = **224**	**AG *n*** = **141**	**GG *n*** = **20**	**AG + GG *n*** = **161**	Adjusted *P* value (additive)^a^^,^^b^	Benjamini-Hochberg false discovery rate *q* (additive)	Adjusted *P* value (dominant)^b^^,^^c^	Benjamini-Hochberg false discovery rate *q* (dominant)
Pulse wave velocity, m/s	7.67 (1.69)	7.73 (1.37)	7.47 (2.43)	7.70 (1.54)	1.00	1.00	.48	0.64
Augmentation index, %	24.55 (25.54)	24.04 (12.85)	21.45 (10.48)	23.71 (28.54)	.28	0.91	.43	0.64
Left ventricular mass index, g/m²	80.31 (25.37)	80.64 (29.01)	79.00 (17.93)	80.37 (27.73)	.73	0.91	.54	0.64
Interventricular septal thickness, cm	0.95 (0.18)	0.95 (0.21)	0.96 (0.23)	0.95 (0.22)	.89	0.97	.92	0.92
Left ventricular posterior wall thickness, cm	0.95 (0.18)	0.96 (0.21)	0.97 (0.22)	0.97 (0.21)	.40	0.91	.27	0.64
Left ventricular end-diastolic diameter, cm	4.63 (0.52)	4.62 (0.44)	4.52 (0.39)	4.61 (0.43)	.76	0.91	.52	0.64
Left ventricular end-systolic diameter, cm	2.63 (0.38)	2.67 (0.40)	2.56 (0.26)	2.66 (0.38)	.62	0.91	.29	0.64
Aortic root diameter, cm	3.05 (0.38)	3.17 (0.22)	2.93 (0.33)	3.13 (0.27)	.68	0.91	.48	0.64
Left atrial diameter, cm	3.10 (0.49)	3.05 (0.38)	3.11 (0.34)	3.06 (0.38)	.60	0.91	.72	0.79
Right ventricular diameter, cm	2.42 (0.29)	2.37 (0.29)	2.44 (0.30)	2.38 (0.29)	.15	0.91	.20	0.64
Left ventricular ejection fraction, %	64.61 (2.97)	64.76 (2.77)	65.44 (1.85)	64.85 (2.68)	.25	0.91	.34	0.64
E/Ea	6.23 (5.34)	7.07 (2.78)	8.11 (11.16)	7.21 (7.19)	.46	0.91	.44	0.64

^a^ Adjusted *P* value from multivariable linear regression under an additive genetic model (effect per minor allele; *STK39:* per G).

^b^ All *P* values are adjusted for age, sex, body mass index, smoking status, fasting plasma glucose, low-density lipoprotein cholesterol, high-density lipoprotein cholesterol, triglycerides, serum creatinine, and thyrotropin.

^c^ Adjusted *P* value for the dominant genetic model comparison (AG + GG vs AA) from multivariable linear regression.

For *ATP2B1* rs2681472, none of the arterial stiffness or echocardiographic traits showed statistically significant heterogeneity across genotypes in either the additive or dominant models. After applying the Benjamini-Hochberg procedure to control the false discovery rate, none of these associations reached statistical significance (all false discovery rate–adjusted *q* > 0.05) (Supplementary Table 2).

## Discussion

In this case-control study of Turkish adults, we interrogated 2 widely studied BP loci—*STK39* rs35929607 (A>G) and *ATP2B1* rs2681472 (C>T)—for association with hypertension status, clinic BP, and secondary cardiovascular traits. We observed that *STK39* rs35929607 was associated with hypertension risk and higher systolic BP in our cohort. In multivariable models, each additional G allele was associated with higher odds of hypertension (AOR, 1.97 [95% CI, 1.21-3.21]) and with higher systolic BP, whereas diastolic BP showed a borderline, nonsignificant increase under the dominant model (*P* = .06; additive *P* = .10). In contrast, *ATP2B1* rs2681472 showed no association with hypertension, systolic or diastolic BP, or the secondary phenotypes; after false discovery rate control, no secondary trait remained statistically significant.

The relationship between hypertension and *STK39* is quite heterogeneous across different populations and single-nucleotide variations. In the first GWAS to implicate this locus, an Old Order Amish GWAS linked *STK39* variants with systolic and diastolic BP.^7^ In 2 population-based Malmö cohorts (Sweden), rs35929607 (A>G) was reported to be associated with prevalent hypertension and with systolic and diastolic BP traits in that setting, with adjusted dominant-model ORs ranging from approximately 1.08 to 1.15. In addition, the risk allele was observed to be associated with increased salt sensitivity.^18^ In rural Pakistan, each G allele of rs35929607 was associated with an increase in systolic BP of approximately 3.07 mm Hg (*P* = .001). After adjustment for potential confounders, however, the association between G allele carriage and hypertension was no longer statistically significant.^19^ A British family-based study, however, reported no association of rs35929607, rs3754777, and rs6749447 with clinic or ambulatory BP, despite clear cis-acting effects on *STK39* expression.^20^ Likewise, a Chinese case-control study observed no association for several *STK39* polymorphisms with hypertension.^21^ In the East Asian population, rs3754777 has been demonstrated to be associated with essential hypertension in central-south Chinese men, while rs3754777/rs6749447 has not been found to be associated with this condition in Korean data.^22^^,^^23^ Consistent with the overall cohort-level findings from the Malmö cohorts (Sweden), in our Turkish sample, *STK39* rs35929607 was associated with increased hypertension risk and higher systolic BP. In logistic regression, the per-allele effect of the G allele on hypertension was moderate (AOR, 1.97 [95% CI, 1.21-3.21]; *P* = .007; *q* = 0.02), and a dominant comparison of carriers (AG + GG) vs noncarriers (AA) also indicated increased risk (AOR, 2.06 [95% CI, 1.19-3.58]; *P* = .01; *q* = 0.02). The GG genotype was rare, resulting in an imprecise but directionally consistent estimate. In linear models, the same allele was associated with higher systolic BP (additive β=+8.02 mm Hg per G allele [95% CI, 2.18-13.87]; *P* = .007; dominant AG + GG vs AA: +9.54 mm Hg [95% CI, 2.65-16.44]; *P* = .007), whereas diastolic BP was nonsignificant after adjustment (additive model *P* = .10; dominant model *P* = .06). Notably, in sex-stratified analyses, the association of *STK39* rs35929607 with hypertension and systolic BP appeared more pronounced in men. In men, the G allele was associated with higher odds of hypertension (additive AOR, 3.22; dominant AOR, 3.23) and higher systolic BP (β=+15.88 mm Hg per allele), whereas in women no statistically significant associations were observed across models. This pattern contrasts with the Malmö cohorts reporting stronger effects in women. In the Malmö cohorts, after stratification by sex, the association remained statistically significant in women but not in men, underscoring potential population-, context-, or sampling-related heterogeneity. Because sex stratification reduces sample size and event counts within each subgroup—especially for the rare GG genotype—precision is reduced; thus, our sex-specific findings should be considered exploratory and require replication. In additional support of functional plausibility, public Genotype-Tissue Expression data indicate that rs35929607 acts as a tissue-specific cis–expression quantitative trait locus for *STK39* in Heart–Left Ventricle, with higher expression in G-allele carriers (median: AA −0.047 [*n* = 271], AG 0.100 [*n* = 156], GG 0.064 [*n* = 23]; *P* = 2.33 ×10^−5^).^24^ Although *STK39*/SPAK is primarily discussed in the context of renal sodium handling, kidney tissues were not among the clinically significant Genotype-Tissue Expression quantitative trait locus signals for this variant; this does not exclude renal regulatory effects, which may be tissue or cell-type specific and underpowered in bulk-tissue resources. We emphasize that expression quantitative trait locus findings do not establish causality; rather, they provide supportive regulatory context in the absence of direct functional assays in our cohort.

A possible explanation for the male-specific association observed in our cohort is that rs35929607 may act within a sex hormone–sensitive pathway. *STK39* encodes SPAK, a kinase that activates NCC and NKCC2 and thereby promotes distal sodium chloride reabsorption. In androgen-responsive lymph node carcinoma of the prostate cells, *SPAK/STK39* has been identified as an androgen-regulated gene, with androgen receptor agonists increasing SPAK expression in a dose- and time-dependent manner.^25^ In rodent kidney, estradiol, progesterone, and prolactin modulate the SPAK-NCC axis by increasing full-length SPAK expression/phosphorylation and NCC phosphorylation, and ovariectomy reverses these effects.^26^ Together, these data support the view that the STK39/SPAK pathway is sensitive to sex steroid signaling. In a predominantly androgenic milieu, a modest rs35929607-related increase in STK39 activity could therefore translate into a relatively larger gain in SPAK/NCC function, enhancing sodium retention, intravascular volume, and BP in men, whereas the same variant might have a smaller or more complex net effect in women because of the concurrent actions of estrogens and progesterone on vascular tone and renal sodium handling. Although these mechanisms have not been tested directly in human kidney or for rs35929607 itself, they provide a biologically plausible framework for our finding that this variant was associated with hypertension and higher systolic BP in men but not in women.

*ATP2B1* encodes PMCA1, a ubiquitous Ca^2+^ efflux pump that is highly expressed in vascular smooth muscle and is essential for maintaining low cytosolic Ca^2+^. Experimental data provide strong evidence that this pathway plays a mechanistic role in BP regulation: partial reduction or knockout of *ATP2B1* in mice raises BP, increases myogenic tone and vasoconstrictor responses, and induces small-artery remodeling.^9^^,^^10^ Consistent with this mechanism, common variants near *ATP2B1*, including rs2681472 and rs17249754, have been reproducibly associated with hypertension and with modest per-allele increases in systolic and diastolic BP (on the order of ≈1 mm Hg and 0.5 mm Hg, respectively) in large European and East Asian meta-analyses.^11^^,^^27^^,^^28^ More recently, *ATP2B1* polymorphisms have also been linked to resistant hypertension in Japanese patients and to an increased prevalence of hypertension in individuals with high metabolic risk scores, further extending the clinical spectrum of this locus.^29^^,^^30^

In our Turkish case-control sample, we did not detect a statistically significant association between *ATP2B1* rs2681472 and hypertension status or clinic BP. The allelic OR for hypertension was 1.18 (95% CI, 0.74-1.87; *P* = .49), and systolic/diastolic BP regression coefficients were small and nonsignificant. These estimates are numerically consistent with the small per-allele effects reported in large consortia, but the wide CIs indicate that our study was underpowered to confirm such modest associations. Thus, our results do not argue against a role for *ATP2B1* in BP regulation; rather, they suggest that rs2681472, if it influences hypertension risk in this population, does so with an effect size too small to be reliably detected in a study of this size.

All hypertensive cases in our study were newly diagnosed and completely treatment naive; current or previous use of antihypertensive drugs was an exclusion criterion. This design minimizes confounding by therapy and makes the observed BP differences more reflective of the underlying physiology, but it also means that case values represent untreated extremes, which can exaggerate genotype-BP gradients compared with population-based cohorts that include longstanding, treated hypertension. In addition, the sex-stratified analyses were based on relatively small subgroups, especially after multivariable adjustment, so the male-specific effect estimates for systolic and diastolic BP are large but imprecise and should be regarded as hypothesis generating rather than a precise quantification of the variant’s impact.

Several limitations should be acknowledged. First, this was a single-center case-control study with a relatively modest sample size, which may limit statistical power and generalizability. Because our cohort primarily represents middle-aged adults with essential hypertension, these findings may not be generalizable to younger or early-onset hypertension cohorts. Second, sex-stratified analyses further reduced the effective sample size, so the sex-specific ORs and regression coefficients have wide CIs and should be interpreted with caution until replicated in larger cohorts. Third, we genotyped only 1 common variant in *STK39* and 1 in *ATP2B1* and did not assess other loci in these pathways or genome-wide, so residual confounding by unmeasured genetic variation cannot be excluded. Although case and control individuals were recruited from the same geographic catchment area at a single center and related individuals were excluded, we did not perform genome-wide ancestry inference (eg, principal components/ancestry-informative markers); therefore, subtle residual population stratification cannot be fully excluded. In addition, structured family history data (eg, hypertension in first-degree relatives) were not available for inclusion as a covariate. Finally, we did not measure *STK39* expression, SPAK phosphorylation, or dietary factors such as salt intake and did not conduct functional experiments; therefore, the present findings, particularly regarding *STK39*, should be interpreted with caution and considered the basis for future replication and mechanistic studies.

In Turkish adults, *STK39* rs35929607 showed an association with hypertension risk and higher systolic BP, whereas *ATP2B1* rs2681472 showed no evidence of association with hypertension, clinic BP, or secondary cardiovascular traits after correction for multiple testing. These findings are biologically plausible; however, larger multicenter replication with refined phenotyping, together with functional studies (eg, expression and pathway activity assays) and fine-mapping, is needed to confirm generalizability and elucidate mechanism.

## Supplementary Material

lmag025_Supplementary_Data

## Data Availability

The data underlying this article will be shared on reasonable request to the corresponding author.
