# Supplementary material for: Association of ATP2B1 and STK39 gene variants with blood pressure levels in patients with essential hypertension
Source: Lab Med. 2026 May 20;57(3):lmag025. doi: 10.1093/labmed/lmag025 (PMC13188984; doi:10.1093/labmed/lmag025)
Supplement: lmag025_Supplementary_Data [file lmag025_supplementary_data.zip › OP-LABM260027_PECorr_CmtAttachmentsFolder_Supplementary_Table_1_v2.docx]

## Supplementary Table 1. Genotype distributions, MAF, and HWE (controls) for STK39 and ATP2B1

| **Gene (SNP)** | **Alleles (forward)** | **Controls: Genotypes (major/het/minor)** | **MAF (controls)** | **HWE p (χ²)** | **HWE p (exact mid-P)** | **Cases: Genotypes (major/het/minor)** | **MAF (cases)** |
| --- | --- | --- | --- | --- | --- | --- | --- |
| STK39 (rs35929607) | A>G | 124 / 60 / 7 | 0.1937 | .94 | 0.91 | 100 / 81 / 13 | 0.2758 |
| ATP2B1 (rs2681472) | C>T | 104 / 76 / 11 | 0.2565 | .55 | 0.64 | 97 / 86 / 11 | 0.2784 |

Alleles are reported on the forward (reference) strand. HWE was evaluated in controls using Pearson’s χ² goodness-of-fit and the exact mid-P test (two-sided). Case genotype distributions and MAF are shown descriptively. MAF = (heterozygotes + 2×minor homozygotes) / (2×N).
