# Supplementary material for: Association of ATP2B1 and STK39 gene variants with blood pressure levels in patients with essential hypertension
Source: Lab Med. 2026 May 20;57(3):lmag025. doi: 10.1093/labmed/lmag025 (PMC13188984; doi:10.1093/labmed/lmag025)
Supplement: lmag025_Supplementary_Data [file lmag025_supplementary_data.zip › OP-LABM260027_PECorr_CmtAttachmentsFolder_Supplementary_Table_2_v2.docx]

## Supplementary Table 2. Association between ATP2B1 rs2681472 genotypes and clinical characteristics in the total study population

|  | **CC (n=201)** | **CT (n=162)** | **TT (n=22)** | **CT+TT (n=184)** | **Adjusted p^a^ (additive)** | **Adjusted p^b^ (dominant)** | **BH–FDR q (additive and dominant)** |
| --- | --- | --- | --- | --- | --- | --- | --- |
| PWV (m/s) | 7.66 ± 1.77 | 7.69 ± 1.50 | 7.83 ± 1.09 | 7.70 ± 1.46 | 0.59 | 0.62 | q > 0.05 |
| AIx (%) | 22.15 ± 24.70 | 25.55 ± 21.19 | 33.14 ± 10.29 | 26.45 ± 20.33 | 0.13 | 0.23 | q > 0.05 |
| LVMI (g/m²) | 77.73 ± 24.86 | 83.99 ± 27.96 | 76.15 ± 24.37 | 83.09 ± 27.61 | 0.08 | 0.02 | q > 0.05 |
| IVS (cm) | 0.94 ± 0.20 | 0.96 ± 0.20 | 0.90 ± 0.16 | 0.96 ± 0.19 | 0.64 | 0.28 | q > 0.05 |
| LVPW (cm) | 0.94 ± 0.19 | 0.98 ± 0.19 | 0.92 ± 0.19 | 0.97 ± 0.19 | 0.41 | 0.16 | q > 0.05 |
| LVEDD (cm) | 4.58 ± 0.46 | 4.68 ± 0.51 | 4.52 ± 0.41 | 4.66 ± 0.50 | 0.24 | 0.15 | q > 0.05 |
| LVESD (cm) | 2.61 ± 0.37 | 2.68 ± 0.40 | 2.69 ± 0.32 | 2.68 ± 0.39 | 0.07 | 0.12 | q > 0.05 |
| Aortic root diameter (cm) | 3.15 ± 1.83 | 3.03 ± 0.41 | 2.92 ± 0.28 | 3.02 ± 0.40 | 0.75 | 0.74 | q > 0.05 |
| LAD (cm) | 3.05 ± 0.41 | 3.14 ± 0.47 | 3.01 ± 0.46 | 3.12 ± 0.47 | 0.18 | 0.08 | q > 0.05 |
| RVD (cm) | 2.40 ± 0.28 | 2.40 ± 0.32 | 2.40 ± 0.18 | 2.40 ± 0.31 | 0.25 | 0.32 | q > 0.05 |
| LVEF (%) | 64.82 ± 2.92 | 64.63 ± 2.73 | 64.22 ± 3.23 | 64.59 ± 2.78 | 0.23 | 0.31 | q > 0.05 |
| E/Ea | 6.87 ± 6.13 | 11.46 ± 47.45 | 6.23 ± 1.74 | 10.85 ± 44.61 | 0.35 | 0.32 | q > 0.05 |

Data are presented as mean ± standard deviation. All P values are adjusted for age, sex, body mass index, smoking status, fasting plasma glucose, low-density lipoprotein cholesterol, high-density lipoprotein cholesterol, triglycerides, serum creatinine, and thyrotropin; P < .05 was considered statistically significant. ᵃ Adjusted p value from multivariable linear regression under an additive genetic model (effect per minor allele; ATP2B1: per T). ᵇ Adjusted p value for the dominant genetic model comparison (CT+TT vs CC) from multivariable linear regression.
